# Experimental Investigation of Mechanical and Fracture Behavior of Parallel Double Flawed Granite Material under Impact with Digital Image Correlation

**DOI:** 10.3390/ma16062263

**Published:** 2023-03-11

**Authors:** Lei Zhang, Zhijun Zhang, Ying Chen, Yong Liu, Xinyao Luo, Bing Dai

**Affiliations:** 1 School of Resource Environment and Safety Engineering, University of South China, Hengyang 421001, China; 2 Hunan Province Engineering Technology Research Center for Disaster Prediction and Control on Mining Geotechnical Engineering, Hengyang 421001, China; 3 School of Physics and Optoelectronic Engineering, Shenzhen University, Shenzhen 518060, China; 4 School of Resource and Safety Engineering, Central South University, Changsha 410083, China

**Keywords:** DIC, double fissures, SHPB, damage characteristics, fractal

## Abstract

During the excavation of underground projects, the rock masses left as the bearing support system are also subjected to dynamic loads from the excavation activities ahead. These rock masses have been damaged and fractured during the initial exposure (dynamic loads) and are subjected to static loads in the subsequent process as the support system. In this study, granite rock samples and specimens with different angles were produced, preloaded with different confining pressure, and under a combination of dynamic and static loading tests using a modified dynamic and static loading system: split Hopkinson pressure bar (SHPB). The peak strain and dynamic modulus of elasticity are weakened by the inclination angle in a similar way to the strength, with the specimens showing an evolutionary pattern from tensile strain to shear damage. The change in the inclination angle of flaws would weaken the dynamic and combined strengths, and a larger inclination flaw results in a significant decrease in its strength. Fractal analysis revealed that the fractural dimension was closely related to the fissure angle and showed a good linear correlation with the strain rate. This study will provide an important security assurance for deep mining.

## 1. Introduction

Rocks are solid materials with natural defects, including joints, fractures, laminations, and even pores, which reduce the dynamic and static strength of the rock to varying degrees [1,2]. With the development of urbanization, more and more underground works (shopping malls, passages, pipelines, and other underground works) have been developed to supplement the urban land [3,4]. The construction of underground works does not excavate all the rocks, but always leaves some of them as part of the support system of the underground works [5,6]. In addition, both mechanical excavation and blasting excavation are processes that use dynamic loads to destroy the original state of the rock [7,8,9]. As a result, the part of the rocks with natural defects is left as the support system, bearing the dynamic loads of the subsequent excavation and static loads (support system). The stability of this part of the rock mass has a deep relationship with the reliability of the engineering structure (support mechanism) [10,11,12].

With the aim of ensuring safety in deep mining and other underground projects, many studies have been carried out on fractured rock masses [13,14,15,16,17,18], and some researchers have studied the factual damage of preflawed rocks in different ways [19,20,21]. As known to all, the loading condition could profoundly affect rock distress, as well as the rock type [22]. Rock specimens exhibit mainly mixed-mode fracture under quasi-static loads dominated by shear [23,24], where they generally fail via tension mode under dynamic loads. It is believed that both static prestress and dynamic load can enlarge the dynamic zone of a rock, but this knowledge is limited for coupled tension tests [25,26,27]. Additionally, in the study of the rock failure problem, the method of numerical simulation is welcomed by many scholars. Compared with others, the method of numerical simulation can not only save time and funds, but also overcome the shortcomings of the test operations, and can fully consider various complex conditions. Many scholars have studied the dynamic load of rock by using particle flow code (PFC), such as AUTODYN software and FLAC3D, to establish a numerical split Hopkinson pressure bar (SHPB) test system. Numerical dynamic tests under different impact velocities were conducted. It showed that there was a clear relationship between the variation of the reflected wave and the stress equilibrium state in the specimens.

Kaiser et al. [28] found that recent analyses support the existence of failure modes that change with increasing confinement—a transition from the axial splitting at unconfined or low confinement to the shear failure at high confinement. The results showed that angle, length, number, and lamination impact the mechanical properties of the fractured rock’s mass under static loading conditions. With the aim of enhancing the support effect of the fissure development area, Deng et al. [29] conducted uniaxial compression tests on a single flaw sandstone of various angles and lengths using acoustic emission devices. The results showed that the maximum axial load-bearing capacity of the specimens decreased with increasing fissure length. In addition, the inclination angle of the prefabricated fissure had some influence on the maximum axial load-bearing capacity of the sandstone, but it was less than the influence of the fissure length on the load-bearing capacity of the specimens. Dou et al. [30,31] performed uniaxial compression tests on sandstones with precast fractures of different inclination angles. The results revealed that the fissure inclination angle had a large influence on the mechanical strength of the fractured rock. With the increase in the fissure inclination angle, the peak strength and elastic modulus of the prefractured rock first decreased and then increased. When the fissure inclination angle was 45° with the direction of the applied axial stress parallel to the loading direction, the initial damage and damage rate of the rock during the fissure evolution were the largest, and the resistance to deformation and damage was the weakest. In addition to the properties of the fissures, the mechanical properties of the rock mass are also influenced by the fissures of different laminae. Lin et al. [32] conducted uniaxial compression tests on a jointed rock mass with two different laminae to investigate the influence of the joint angle and rock bridge angle on the mechanical behavior and damage process of the laminated rock mass. The results indicated that the peak strength of the rock mass was not only related to the joint and bridge pinch angles but was also influenced by the laminae, and the rock mass damage was mainly caused by the extension of cracks in the low-strength laminae. In addition, the number of fissures also affects the mechanical properties of the rock mass under static loading.

The above studies are all about the mechanical properties and damage characteristics of fractured rock under static loads. However, during the excavation of underground projects, fractured rock is often subjected to coupled dynamic and static loads. Regarding this problem, some scholars have also carried out relevant studies [4,18,33,34].

Wang et al. [35] carried out SHPB impact tests with boundary conditions on fractured rocks and found that the boundary conditions play an important role in the rock fracture process. In addition, the fracture angle also affects the dynamic strength of the fractured rock mass. Dai et al. [36] carried out an experimental study of dynamic and static coupling loading using a fractured rock mass. The result revealed that the coupling strength initially decreased with increasing fracture inclination, reached a minimum at approximately 45°, and then increased with increasing fracture inclination. Li et al. [37] used an improved SHPB device to study the energy consumption of sandstone samples with prefabricated fissures and holes under combined dynamic and static loading. Weng et al. [38] used rock-like specimens with single fractures and an MTS-793 test system to study the energy damage characteristics of rock loaded with a low strain rate under a one-dimensional dynamic and static combined loading. Tao et al. [39] studied the stress concentration and energy dissipation around the cavity of the rock sample under the combined action of static load and stress wave, and the results demonstrated that a higher prestatic load is more likely to cause cavity damage. To a certain extent, the dissipated energy of the rock reflected the damage inside the rock.

In addition to the mechanical properties, studies of fracture development under dynamic and static loadings also yielded interesting phenomena. Liu et al. and Yan et al. [40,41] found that the length of the FPZ (fracture development zone) was significantly longer under dynamic loading than under static loading, and the length of the FPZ generally increased with an increase in strain rate, showing a clear rate dependence. Furthermore, the fracture inclination angle had a significant effect on the fracture development and damage mode of the rock mass under dynamic loading. Ai et al. [42] conducted impact tests on coal bodies containing vertical and horizontal joints at different impact velocities. The results showed that the joint orientation had a significant effect on the crack expansion path. Furthermore, numerical simulations demonstrated that the central crack development accelerates with the increase in impact velocity.

**Figure 1:**
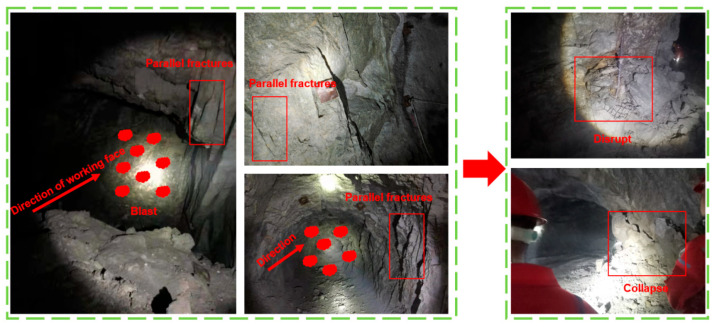
Hazards caused by dynamic and static coupling loads.

The existing studies have been carried out on the dynamic strength and fracture development of single fractured rock masses, but most rock masses contain many fractures in the natural environment. In addition, the rocks excavated under dynamic loads continue to bear the dynamic loads that were generated from the excavation blast at the front and affected by both dynamic and static loads (Figure 1). The realization of advanced optical instruments is instrumental in exploring many of the details in the break process, and is particularly promising for the research of damage and stability in deep mining. In these studies, the technique was used to collect the data during the test. In this paper, the bi-parallel fractured rock mass will be used for dynamic and static loading tests. The damage process and the variation of the strain field of a bi-parallel fractured rock under different dip angles and envelope pressure conditions are discussed.

The structure of this paper is as follows: Section 2 presents the experimental methodology, such as the materials, equipment and the data processing methods. Section 3 demonstrates the experimental results, including the dynamic stress-strain curves of specimens with different angles, the influences of flaw angle and axial pressure on the dynamic and combined strengths, peak strain and modulus of elasticity. Section 4 discusses the influences of flaw angle and axial pressure on the damage process, especially on the evolution characteristics of the strain field at the crack end. Section 5 illustrates the strain field evolution laws of specimens under different axial pressures. Section 6 discusses the final damage pattern of granite specimens at the same fracture inclination, influenced by the magnitude of the applied static load. Section 7 concludes the whole study.

## 2. Experimental Methodology

### 2.1. Specimen Preparation

**Figure 2:**
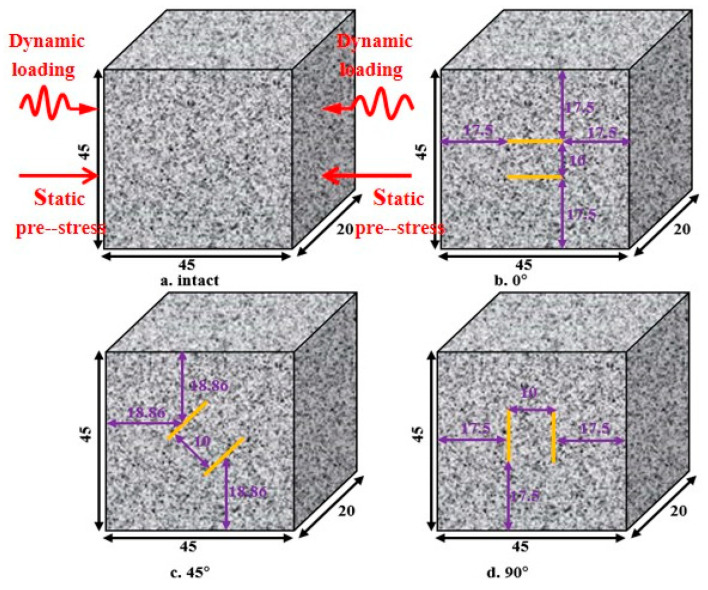
Granite specimen.

First, the rock samples were cut from the rock mass in the undisturbed part. In order to obtain a clearer observation of crack evolution, a single granite with good geometric integrity and uniformity was cut to obtain prismatic samples with a height, width and thickness of 45, 45 and 20 mm, respectively, which proved the feasibility and effectiveness of prismatic rock samples in an SHPB test. Then, the high-pressure water jet cutting machine was used to cut the rock specimens into length, width and thickness of 10 mm, 1 mm and 1 mm parallel cracks at different angles (0°, 45° and 90°). The geometry of the precut specimens is shown in Figure 2. After cutting the specimen rocks, the loading ends of all specimens were polished according to the standards of the International Society of Rock Mechanics (ISRM). The samples with different flaws were divided into 6 groups. The detailed geometric dimensions and mechanical parameters of each group are shown in Table 1. Among them, Int represents the complete sample, and S_0_-flaw0°-1 represents the first sample of a 0° fracture sample at 0% UCS.

### 2.2. Experimental Apparatus and Testing Procedure

**Figure 3:**
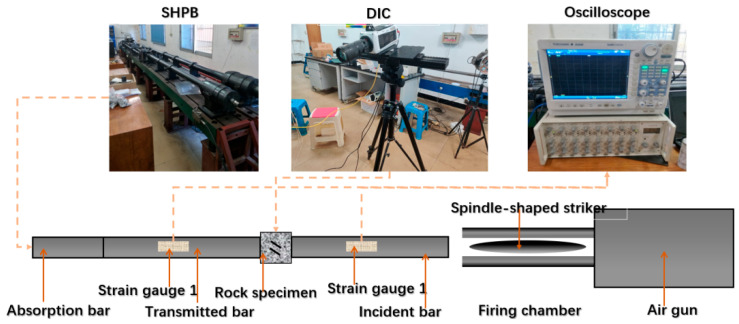
SHPB test device.

In this paper, the one-dimensional dynamic and static combined loading test is based on the SHPB device system of Central South University, Changsha, China, as shown in Figure 3. The test system includes a stress wave generation device, a stress transfer mechanism, axial static pressure loading parts, confining pressure (static pressure) loading parts and a data acquisition and processing device. During the test, the axial static pressure device is first used to apply the axial pressure required for the test to the sample, and then the stress wave generating device is started. The alloy bullet impacts the elastic rod to generate a sinusoidal-shaped loading stress wave, and then the stress wave propagates along the incident rod. Transmission and reflection waves occur on the contact surfaces of the rock sample and the elastic rod. The transmitted stress wave continues to pass forward to the transmission rod, and the reflected wave returns to the incident rod. The transient stress wave signal in the rod is collected by the strain gauge attached to the incident and transmission rods, then the signal is transmitted to the micro-computer system for processing, and then the various parameters of the sample are tested. 

The digital image correlation (DIC) technique is a noncontact measurement that can obtain the full field displacement and strain distribution of the sample surface by matching the positions of the speckle before and after deformation in the region of interest (region of interest, ROI). With the help of a high-speed camera, this study aims to obtain the crack growth process and deformation field distribution at a higher resolution. The ROI region is composed of many subsets, which contain speckles of different quantities and sizes, making each subset unique. When the specimen surface was deformed, the subsets in the deformed and reference images were matched by the analysis of DIC, and finally a grid containing displacement and strain information relative to the reference image were obtained.

The displacement field is calculated by the following formula [43]:

(1) $$\left\{\begin{array}{c} x_{i}^{\prime }=x_{i}+u+\frac{\partial u}{\partial x}\Delta x+\frac{\partial u}{\partial y}\Delta y\\ y_{i}^{\prime }=y_{i}+v+\frac{\partial v}{\partial x}\Delta x+\frac{\partial v}{\partial y}\Delta y \end{array}\right.$$

where u and v are displacement components of the center point along the *x*- and *y*- direction, respectively; $x_{i}$, $y_{i}$, $x_{i}^{\prime }$ and $y_{i}^{\prime }$ are the coordinate components of each point; $\frac{\partial u}{\partial x}$, $\frac{\partial u}{\partial y}$, $\frac{\partial v}{\partial x}$ and $\frac{\partial v}{\partial y}$ are the first-order displacement gradients of the reference subset.

In this study, as DIC technology was used to obtain the strain field on the rock sample surface, artificial speckle should be created on the sample observation surface in advance. First, a layer of white matte paint was uniformly sprayed on the test surface to form the background color, and the random speckle was created with black matte paint. A high-speed camera (V711, Phantom, Vision Research, Wayne, NJ, USA) was used to photograph the sample surface to analyze the changes in the strain field. In this experiment, the shooting resolution of the high-speed camera was 256 × 256, the shooting speed was 79,166 fps (i.e., a frequency of 153,000 frame/s) and the TTL signal was synchronously triggered by connecting with the hyperdynamic strain gauge. The shooting angle of the two cameras was not more than 60°, and the distance between the camera and the sample was approximately 1.5 m.

### 2.3. Data Processing Method

For fractured rock samples, the cracks in the sample will inevitably have a certain influence on the propagation of stress waves. Therefore, before the analysis of the results, it is necessary to clarify the effectiveness of the SHPB test in studying the dynamic mechanical properties of fractured rock samples. In the SHPB test, only when the stress state in the sample reaches the stress balance before the failure is the dynamic strength data valid. The SHPB test system needs to meet two basic assumptions: (1) One-dimensional stress wave hypothesis, the stress wave on the bar in the SHPB system is assumed to be a one-dimensional stress wave and the specimen is also in a one-dimensional loading state, ignoring the wave form dispersion effect. (2) In the SHPB test, the stress field and strain field inside the specimen should be ensured to be uniform, so as to avoid premature failure before the specimen reaches the peak stress, especially for the specimen with cracks.

Based on the one-dimensional stress wave theory, the stress ($\sigma_{s}$), strain (ε) and strain rate (${\dot{\varepsilon }}_{s}$) histories of specimens can be derived using three waves [44]. According to the references [45] and based on the 1D wave theory, the average strain $\varepsilon_{s}\left(t\right)$, strain rate ${\dot{\varepsilon }}_{s}\left(t\right)$ and stress $\sigma_{s}\left(t\right)$ in the specimen can be derived as follows [46]:

(2) $$\left\{\begin{array}{c} \varepsilon_{s}\left(t\right)=-\frac{2C_{e}}{L_{s}}\int_{0}^{t}\varepsilon_{R}\left(t\right)dt\\ {\dot{\varepsilon }}_{s}\left(t\right)=-\frac{2C_{e}}{L_{s}}\varepsilon_{R}\left(t\right)\;\\ \sigma_{s}\left(t\right)=\frac{E_{e}A_{e}}{A_{s}}\varepsilon_{T}\left(t\right)\;\; \end{array}\right.$$

where $\varepsilon_{R}(t),\;\varepsilon_{T}(t),\;A_{e}$, $C_{e}$, and $E_{e}$ are the reflected strain, transmitted strain, cross-sectional area, P-wave velocity and Young’s modulus of the elastic bar, respectively; $A_{s}$ and $L_{s}$ are the cross-sectional area and length of the specimen, respectively.

## 3. Results

### 3.1. Stress Equilibrium

**Figure 4:**
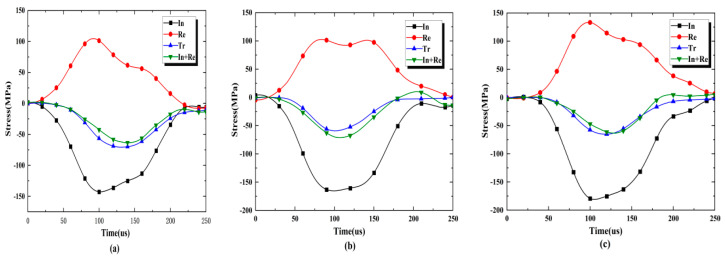
Dynamic stress equilibrium in specimens: (**a**) S_A_-flaw0°-2, (**b**) S_B_-flaw45°-1 and (**c**) S_C_-flaw90°-2.

For the accuracy of the test, the dynamic stress balance of the test must be resolved. It can be seen from Figure 4 that the dynamic loading force at both ends of the specimen is basically equal during the SHPB dynamic loading process. The curve variation of the superposition of the incident stress and the reflected stress (In + Re) is basically the same as that of the transmission stress (Tr), indicating that each test specimen has reached the dynamic stress balance during the loading process. Under the stress balance, because the difference in loading force between the two ends of the specimen is small, the inertial force caused by the inertial effect can be ignored, indicating that the specimen can achieve and maintain the dynamic stress balance condition during the dynamic loading process, thus verifying the validity of the test results.

**Figure 5:**
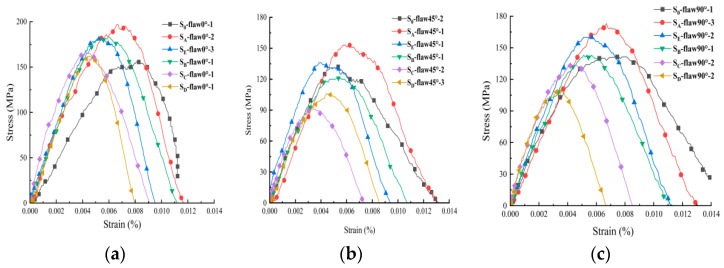
Dynamic stress-strain relationship under different axial static pressures for: (**a**) specimens with 0° flaw; (**b**) specimens with 45° flaw; (**c**) specimens with 90° flaw.

Figure 5 shows that the dynamic stress-strain curve changes from linear to nonlinear before the peak stress, which can be roughly divided into a micro-crack compaction stage, an elastic deformation stage and a post-peak failure stage. The unloading stage of impact stress after peak stress is the stage where dynamic strain increases to the end of impact stress unloading. The reason is that the rock is in the elastic deformation process when the impact disturbance occurs after the internal micro-cracks of the rock are compacted, resulting in the linear growth of the dynamic stress-strain curve. With the increase in impact stress, the micro-cracks in the rock germinate, expand and penetrate and plastic deformation occurs. Especially when the stress reaches the yield stress of the rock, the dynamic stress-strain curve enters the nonlinear stage. The reason why dynamic strain increases at the end of unloading is that in the process of disturbance impact, local damage occurs inside the rock, and the elastic energy stored inside is released more, which is not enough to resist the compressive strength caused by impact stress.

### 3.2. Strength and Deformation Properties

**Figure 6:**
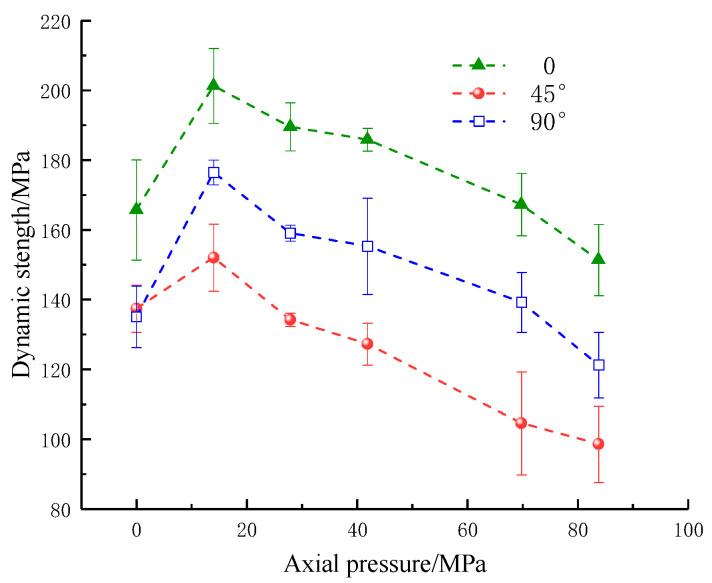
Dynamic strength of specimens with an artificial flaw under different axial prestresses.

**Figure 7:**
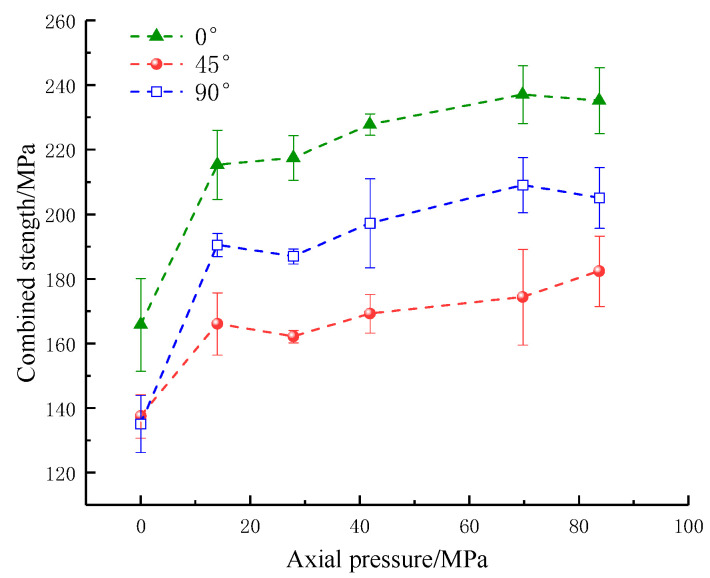
Combined strength of specimens with an artificial flaw under different axial prestresses.

As shown in Figure 6 and Figure 7, with the increase in the applied static axial pressure, the dynamic strength showed an increasing trend and then a decreasing trend, reaching its maximum at an axial pressure of 14 MPa (10% UCS). The combined strength shows an overall increase at 0–14 MPa (0–10% UCS) axial pressure. When the axial pressure is from 14 MPa to 69.8 MPa (10–50% UCS), the strength exhibits a slight increase at 27.9 MPa (20% UCS) axial pressure. Afterward, the combined strength increases slowly in steps and decreases when the axial pressure is from 69.8 MPa to 83.8 MPa (50–60% UCS). Under a certain axial compression, the dynamic strength and combined strength of rock samples first decrease and then increase with the increase in fracture angle, which indicates that the changes in the parallel double fissure inclination angle can weaken the dynamic strength and combined strength, consistent with the results of numerous studies.

The analysis showed that the change in the inclination angle influenced the distribution of the internal stress field of the rock during the impact of the specimen. Macro-scopically, this change manifested as a weakening of dynamic and combined strengths. For larger fracture inclination angles, the strength reduction was significant. According to the theory of fracture mechanics, the stress level required for a prefabricated fracture inclination angle of (90°−φ)/2 is minimal for the same effective shear force, thus explaining the phenomenon of the minimum strength of the 45° fracture specimens. To a certain extent, the axial pressure induces the closure of micro-fractures within the rock, increasing the dynamic and combined strengths. When the axial pressure gradually increases, the excessive static pressure causes the sprouting and expansion of the internal micro-fractures, decreasing the residual bearing capacity under dynamic loading. However, the increase in axial pressure causes the combined loading of the rock to show intensified characteristics. When the applied static pressure is too large and intensifies the microfracture expansion within the rock, the decrease rate of dynamic strength increases, showing a decrease in the combined strength.

**Figure 8:**
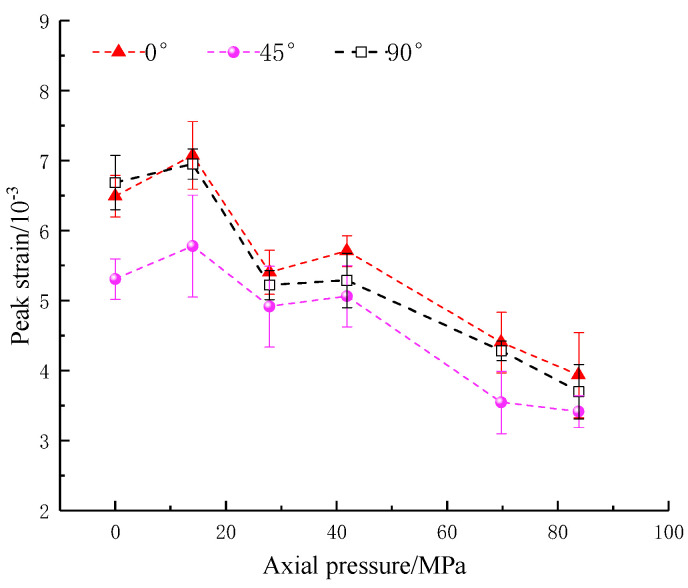
Peak strain of specimens with an artificial flaw under different axial prestresses.

**Figure 9:**
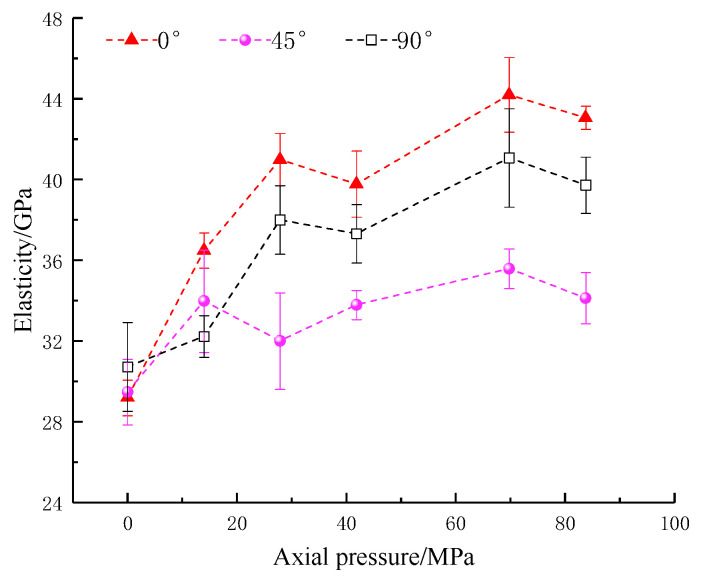
Elasticity of specimens with an artificial flaw under different axial prestresses.

The strain and modulus of elasticity are important indicators of the deformation characteristics of the rocks. The relationship between peak strain, dynamic modulus of elasticity and axial pressure is shown in Figure 8 and Figure 9. It can be seen that the weakening of the peak strain and dynamic elastic modulus by the inclination angle is similar to that of the strength; both of them first decrease and then increase with the increases in the crack angle. The peak strain shows an overall increasing trend and then a decreasing trend as the applied axial static pressure increases. The reason for its stepwise decrease is that the increase in the axial pressure reduces the remaining deformation of the rock. At the axial pressure of 27.9 MPa (20% UCS), the dynamic modulus of elasticity at different fracture inclinations of the precast specimens shows an increase with axial static pressure. At axial pressures of 0~69.8 MPa (0~50% UCS), the overall trend increases step by step, with inflection points at 27.9 MPa (20% UCS) and 41.9 MPa (30% UCS) and a decreasing trend at 69.8–83.8 MPa (50–60% UCS). The reason for the rise in the dynamic modulus of elasticity is similar to that of the combined strength and is related to the compression-density effect of the axial pressure. The decrease at high axial pressures (50–60% UCS), on the other hand, is due to the larger loads making the rock enter an earlier state of damage, weakening its effective load-bearing capacity in the dynamic impact loading and leading to a decrease in the dynamic modulus of elasticity. In this process, the above-mentioned information indices produced a significant turnaround in the trend at axial pressures of 27.9 MPa (20% UCS) and 41.9 MPa (30% UCS). Therefore, the measurement indices of the parallel double fracture specimens at 20–30% static axial pressures should be noted.

## 4. Damage Process

Due to the different types and sizes of subsurface rockwork disturbances, it is necessary to find the influence of dynamic impact loading on the mechanical properties of rocks. In damage characteristics, there is also a lack of findings on dynamic monitoring of crack extension and full-field strain measurements, especially on the evolution characteristics of the strain field at the crack end. Therefore, the damage process was analyzed in detail in this study. Due to space limitations, only the test results for 10%, 30% and 60% axial pressure were analyzed.

### 4.1. The Damage Process of the Specimen with Different Parallel Bi-Flaws at the 10% Axial Pressure

**Figure 10:**
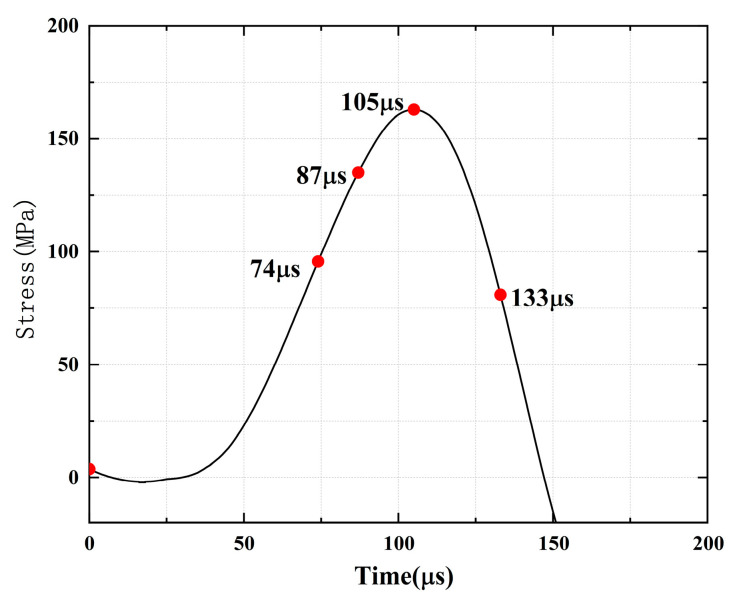
Stress-time curve of a typical specimen with a 0° flaw (S_A_-flaw0°-1).

**Figure 11:**
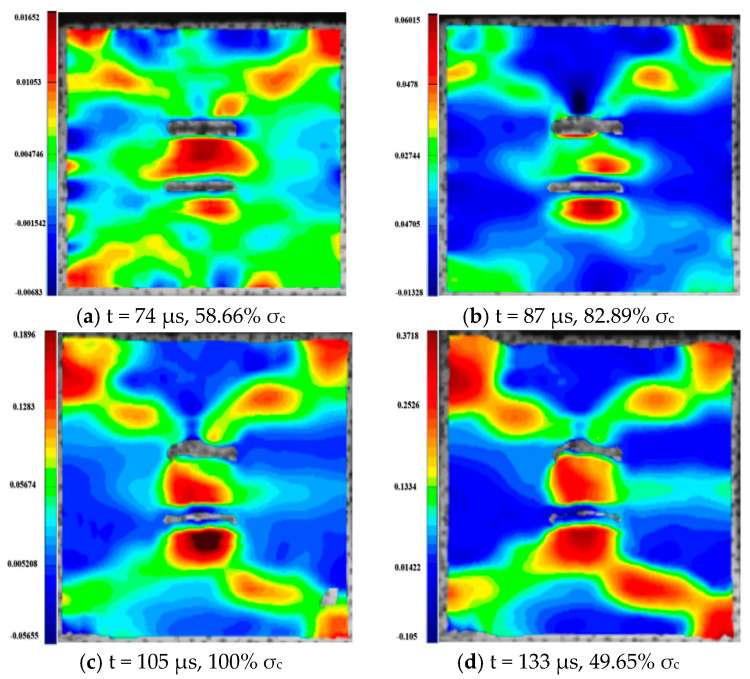
Typical damage process of a rock specimen with a 0° flaw (S_A_-flaw0°-1).

**Figure 12:**
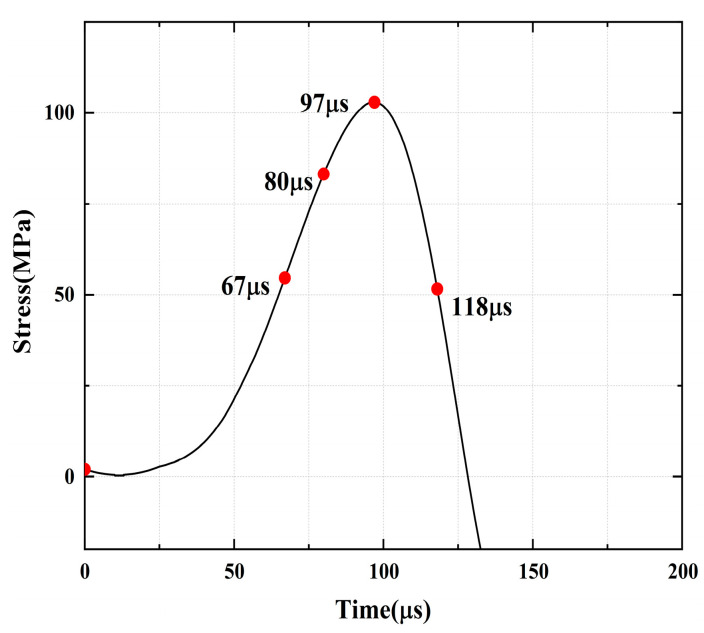
Stress-time curve of a typical specimen with a 45° flaw (S_A_-flaw45°-3).

**Figure 13:**
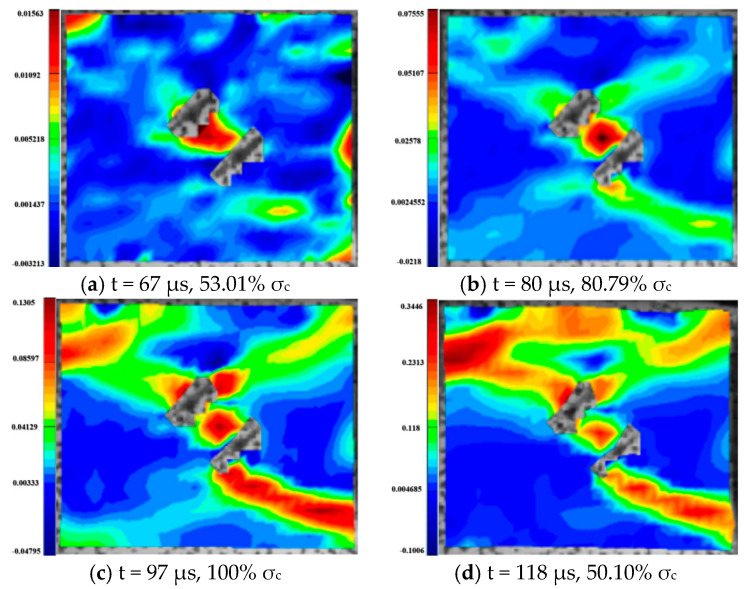
Typical damage process of a rock specimen with a 45° flaw (S_A_-flaw45°-3).

**Figure 14:**
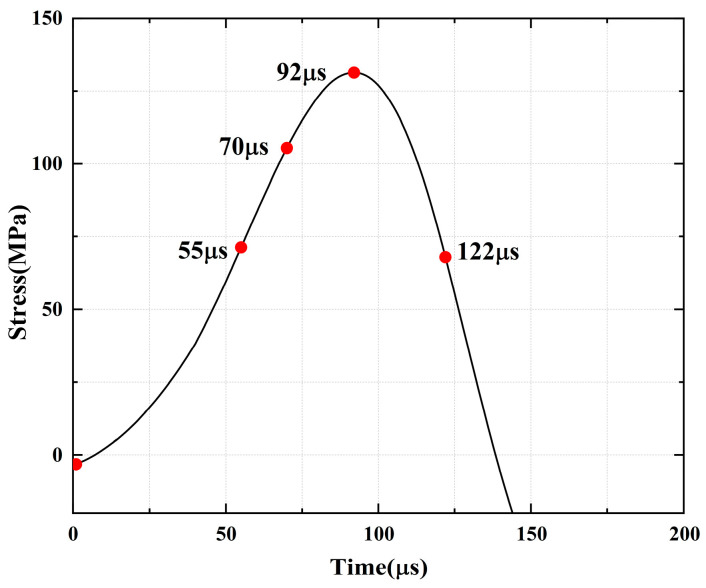
Stress-time curve of a typical specimen with 90° flaw (S_A_-flaw90°-2).

**Figure 15:**
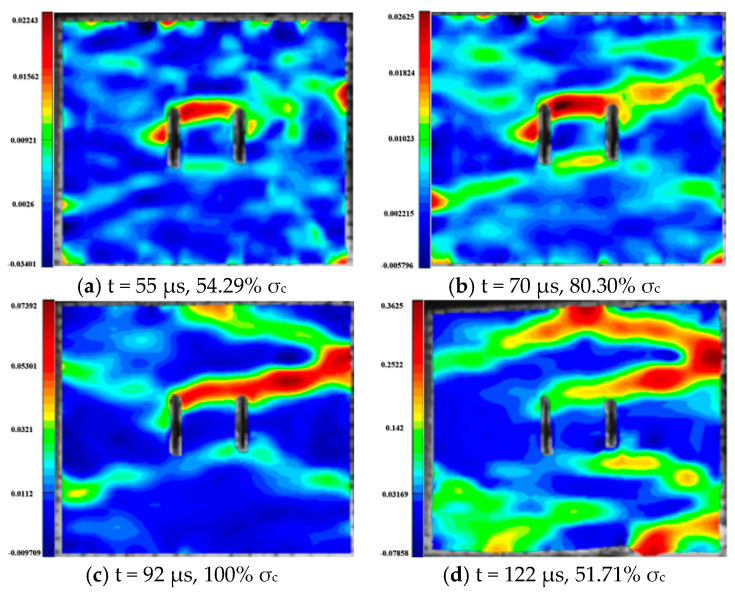
Typical damage process of a rock specimen with a 90° flaw (S_A_-flaw90°-2).

Figure 10, Figure 11, Figure 12, Figure 13, Figure 14 and Figure 15 show the stress-time curve and damage process of the specimens with 0°, 45°, 90° parallel bi-flaws at the 10% axial pressure, respectively.

For the rock specimens with 0° bi-flaws, the stress field and crack propagation mode gradually change under the loading of a static pressure of 10% UCS. We selected four typical stress fields in the loading process and analyzed their changes, as shown in Figure 10. Figure 11a shows that large TSZs (tensile strain zones) have been developed in the middle, upper and lower parts of the parallel bi-flaws, in the upper left and upper right corners, and in the lower left corner. In Figure 11b, the upper part penetrated the upper flaw, forming two oblique SSZ (shear strain zones). Figure 11c reveals that the upper and lower parts of the flaws have been partially destroyed, and the TSZ above and below the flaws have developed into an “X”-shaped shear strain zone centered on the flaws. In addition, the SSZ in Figure 11d penetrated each other, and the central TSZ formed a TS (tensile strain) crack.

For the rock specimens with 45° bi-flaws, the stress field and crack propagation mode gradually change under the loading of a static pressure of 10% UCS. We selected four typical stress fields in the loading process and analyzed their changes, as shown in Figure 12. During loading, a large tensile strain deformation occurred in the bridge region of the parallel bi-flaws (Figure 13a). At the same time, TSZ also appeared in the upper left and right ends and also in the lower part of the specimen. At the end of the flaws in Figure 13b, both inverse and along the SSZ fissure direction appear, and the TS in the center continues to increase. In Figure 13c, the SSZ extended to the upper diagonal of the specimen, while the strain zone extending to the upper diagonal developed horizontally in Figure 13d, forming a TSZ.

For the rock specimens with 90° bi-flaws, the stress field and crack propagation mode gradually changed under the loading of a static pressure of 10% UCS. We selected four typical stress fields in the loading process and analyzed their changes, as shown in Figure 14. In Figure 15a, TSZ is generated at the upper end of the parallel bi-flaws during loading. In Figure 15b, the TS (tensile strain) continues to increase in the TSZ. In Figure 15c, the TSZ has extended to the right-hand boundary, and Figure 15d shows that the strain zone extends to the middle of the top of the specimen and develops horizontally, forming a TS crack and causing damage to the specimen.

### 4.2. The Damage Process of the Specimen with Different Parallel Bi-Flaws at the 30% Axial Pressure

**Figure 16:**
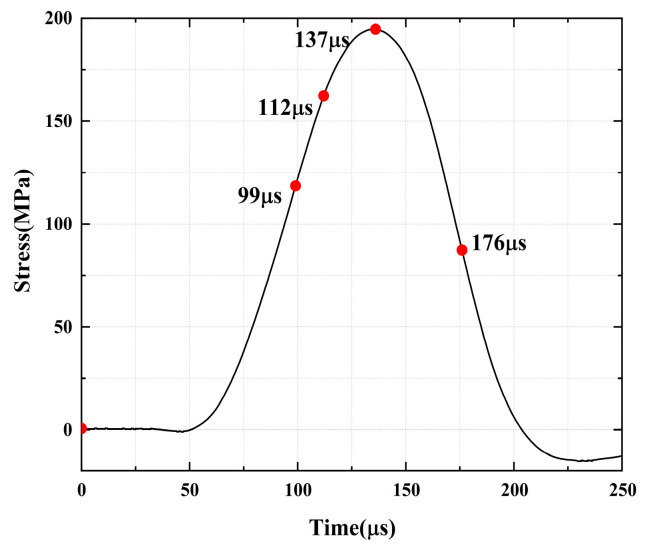
Stress-time curve of a typical specimen with a 0° flaw (S_B_-flaw0°-2).

**Figure 17:**
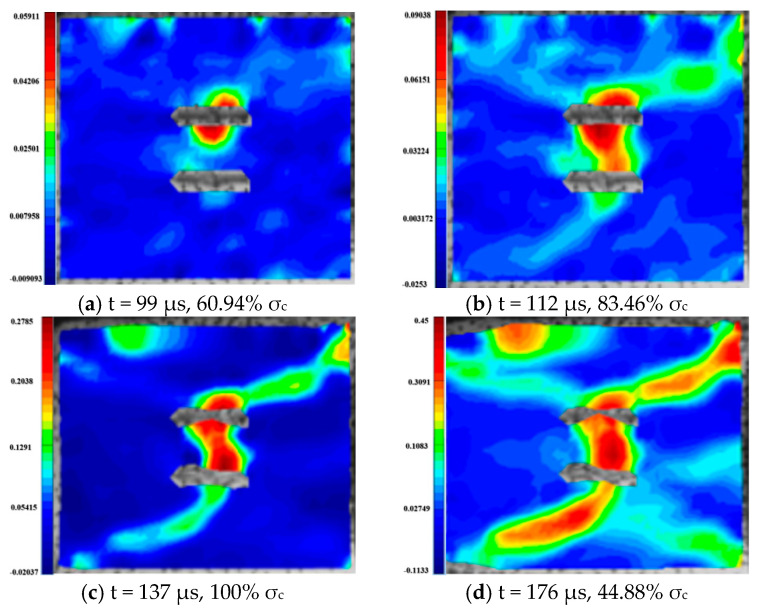
Typical damage process of a rock specimen with a 0° flaw (S_B_-flaw0°-2).

**Figure 18:**
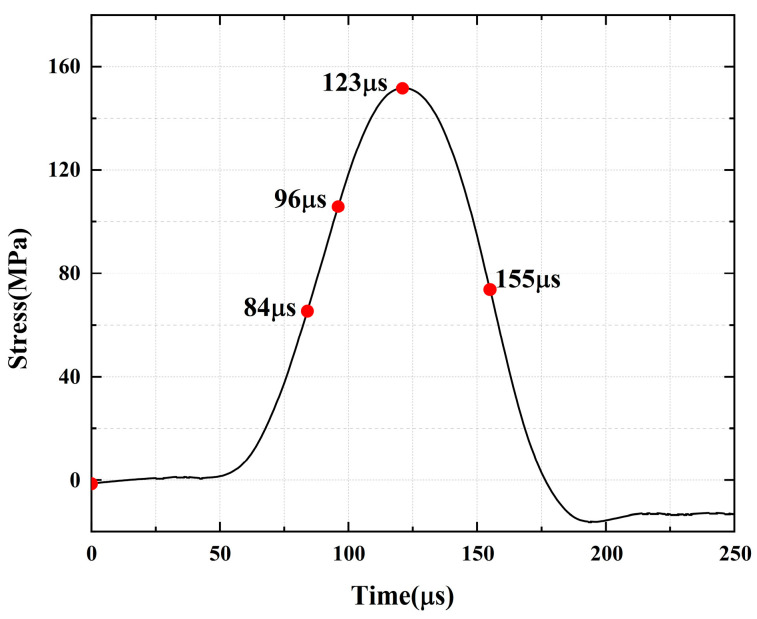
Stress-time curve of a typical specimen with a 45° flaw (S_B_-flaw45°-3).

**Figure 19:**
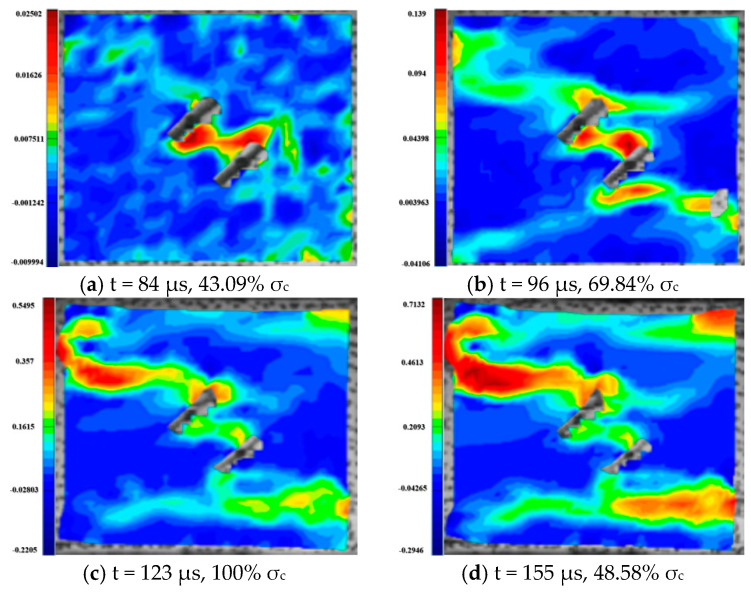
Typical damage process of a rock specimen with a 45° flaw (S_B_-flaw45°-2).

**Figure 20:**
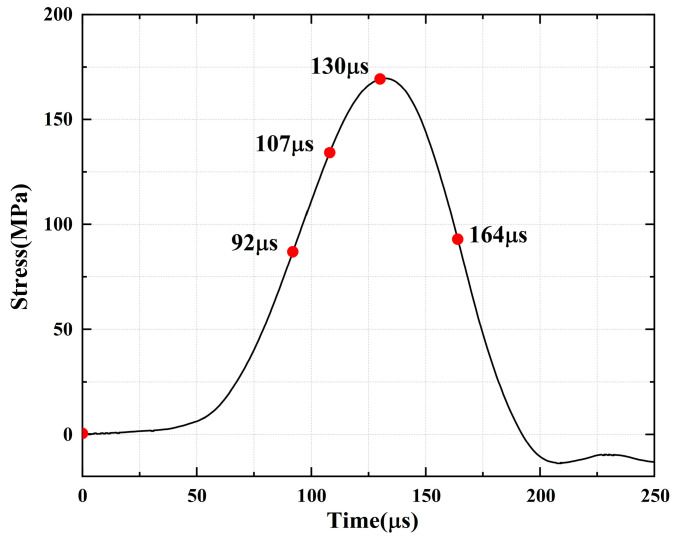
Stress-time curve of a typical specimen with a 90° flaw (S_B_-flaw90°-3).

**Figure 21:**
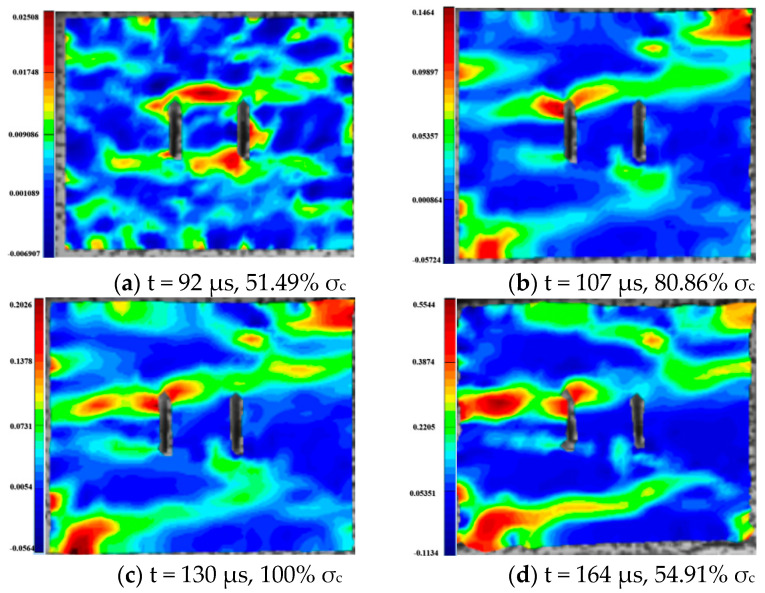
Typical damage process of a rock specimen with a 90° flaw (S_B_-flaw90°-2).

**Figure 22:**
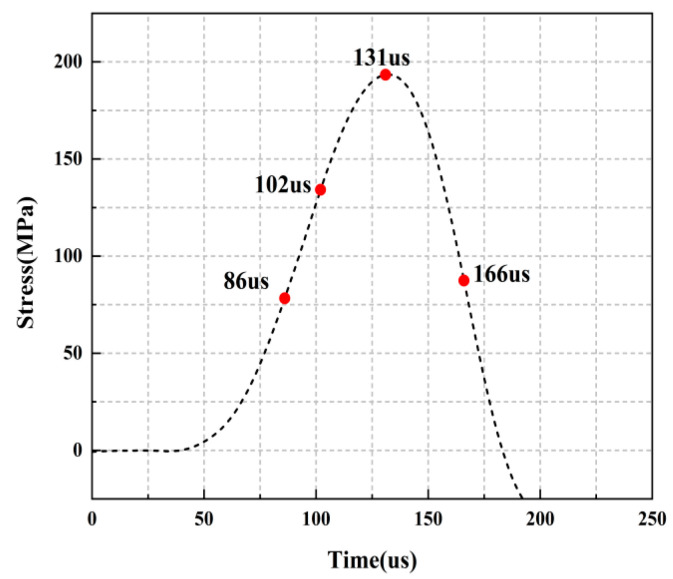
Stress-time curve of a typical specimen with a 0° flaw (S_E_-flaw0°-2).

Figure 16, Figure 17, Figure 18, Figure 19, Figure 20, Figure 21 and Figure 22 show the stress-time curve and damage process of the specimens with 0°, 45° and 90° parallel bi-flaws at the 30% axial pressure, respectively.

For the rock specimens with 0° bi-flaws, the stress field and crack propagation mode gradually change under the loading of static pressure of 30% UCS. We selected four typical stress fields in the loading process and analyzed their changes, as shown in Figure 16. Figure 17b reveals that the upper flaw penetrates the upper right corner to form a shear strain crack, generating a larger TSZ in the middle. The lower part of the lower flaw begins to develop a lower strain, and partial shear damage develops, as shown in Figure 17c. In Figure 17d, two shear cracks extend to the diagonal, connecting the ends of the flaws, which eventually cause damage to the rock.

For the rock specimens with 45° bi-flaws, the stress field and crack propagation mode gradually change under the loading of static pressure of 30% UCS. We selected four typical stress fields in the loading process and analyzed their changes, as shown in Figure 18. As shown in Figure 19a, a larger TSZ appears on the rock bridge with a smaller inverse TSZ at the outer end of the flaw. In Figure 19b, the inverse TSZ develops and extends diagonally towards the upper left and lower right, along with subshear strain cracks towards the flaw direction. Figure 19c shows that the diagonal shear damage pattern developed through the parallel bi-flaws. Eventually, the specimen was damaged by a parallel TSZ developing at the upper end, as shown in Figure 19d.

For the rock specimens with 90° bi-flaws, the stress field and crack propagation mode gradually change under the loading of static pressure of 30% UCS. We selected four typical stress fields in the loading process and analyzed their changes, as shown in Figure 20. In Figure 21a, two TSZ were formed at the upper and lower ends of the two flaws and developed toward the gauge. Figure 21b shows that the TSZ in the upper part expands diagonally to form the SSZ. Furthermore, the strain zone at the lower end developed in the lower left corner to form the SSZ. In Figure 21c, a primary shear strain formed in the lower part, and a shear crack developed in the upper SSZ next to the upper left corner. Finally, the damage was caused by two shear cracks and a tensile crack, as shown in Figure 21d.

### 4.3. The Damage Process of the Specimen with Different Parallel Bi-Flaws at the 60% Axial Pressure

**Figure 23:**
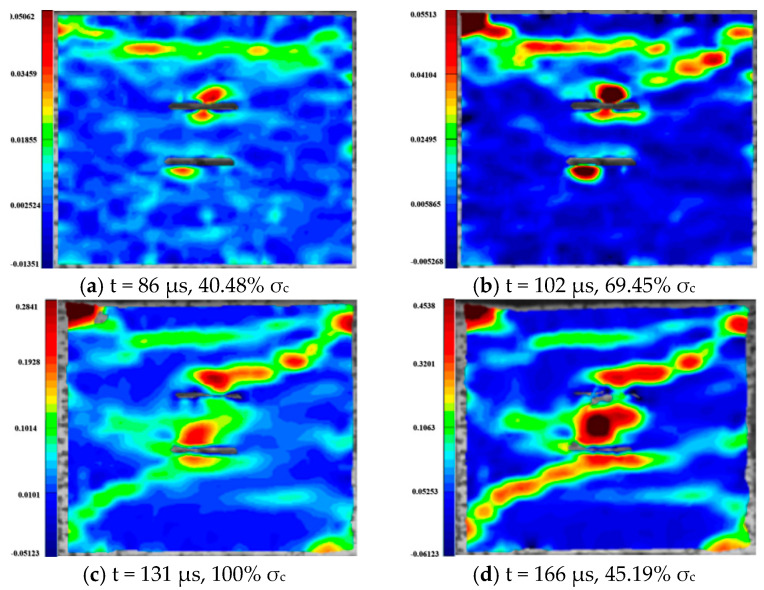
Typical damage process of a rock specimen with a 0° flaw (S_E_-flaw0°-2).

**Figure 24:**
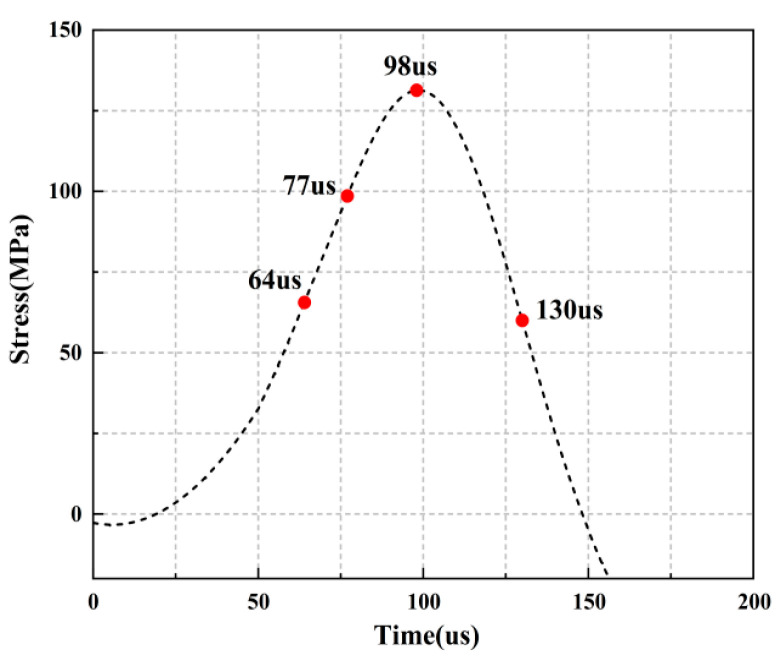
Stress-time curve of a typical specimen with a 45° flaw (S_E_-flaw45°-3).

**Figure 25:**
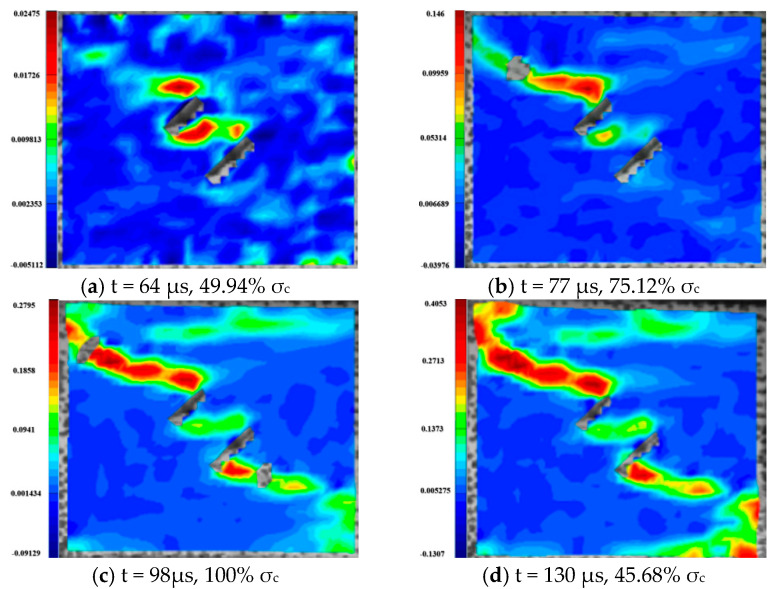
Typical damage process of a rock specimen with a 45° flaw (S_E_-flaw45°-3).

**Figure 26:**
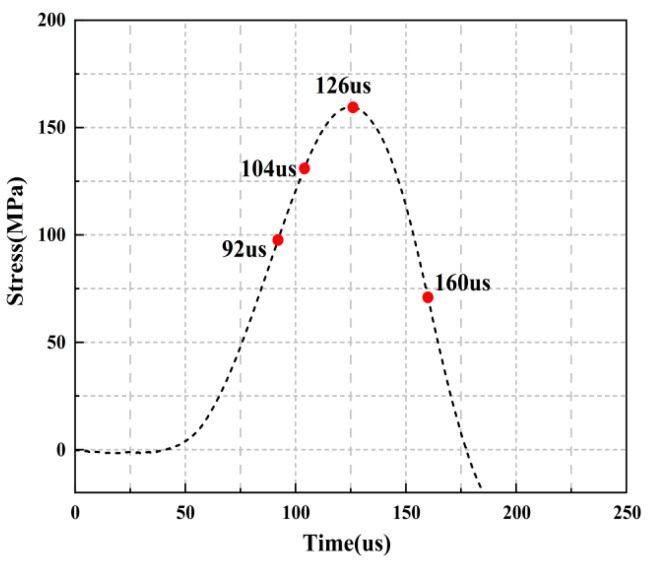
Stress-time curve of a typical specimen with a 90° flaw (S_E_-flaw90°-1).

Figure 23, Figure 24, Figure 25 and Figure 26 show the stress-time curve and damage process of the specimens with 0°, 45° and 90° parallel bi-flaws at the 60% axial pressure, respectively.

For the rock specimens with 0° bi-flaws, the stress field and crack propagation mode gradually change under the loading of a static pressure of 60% UCS. We selected four typical stress fields in the loading process and analyzed their changes, as shown in Figure 22. Figure 23a shows that a TSZ first develops in the upper part, then develops in both the upper and lower parts. As shown in Figure 23b, the upper TSZ further develops into a SSZ towards the upper left corner. In Figure 23c, the TSZ in the lower flaw and center of the bi-flaws also starts to develop, and an SSZ develops toward the lower left corner. Afterward, the SSZ in Figure 23d penetrates both ends and is accompanied by the final damage caused by the newly developed shear cracks at the upper and lower ends.

For the rock specimens with 45° bi-flaws, the stress field and crack propagation mode gradually change under the loading of a static pressure of 60% UCS. We selected four typical stress fields in the loading process and analyzed their changes, as shown in Figure 24. From Figure 25a, it can be seen that a large TSZ is formed in the bridge area of the parallel bi-flaws. At the upper end of the upper flaw, a TSZ is formed in the anticlinal direction. Figure 25b shows that the anticlinal strain zone continues to develop along the diagonal, forming a smaller strain zone in the upper flaw along the fracture direction and in the lower part of the lower flaw. In Figure 25c, the end of the flaw is destroyed, and the shear strain propagates through the specimen along the loading direction. The main shear crack and a horizontal TSZ in the upper part are formed, which eventually lead to the shear damage, as shown in Figure 25d.

**Figure 27:**
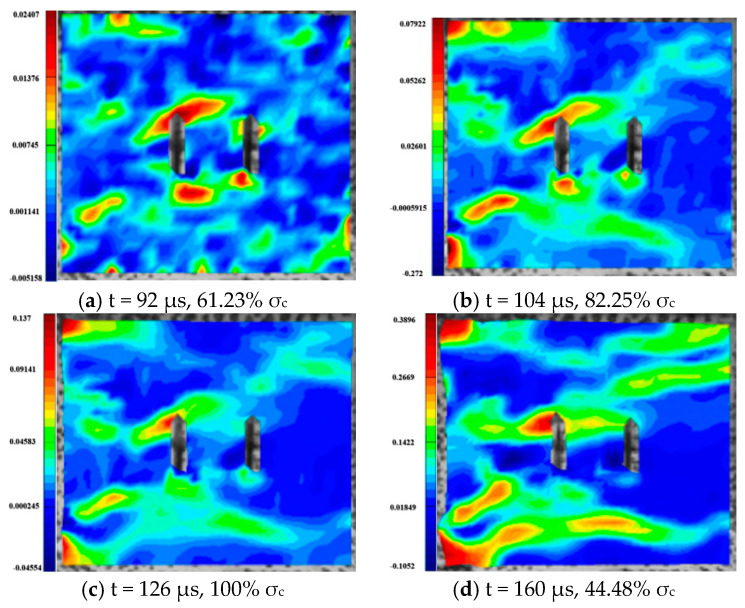
Typical damage process of a rock specimen with a 90° flaw (S_E_-flaw90°-1).

For the rock specimens with 90° bi-flaws, the stress field and crack propagation mode gradually change under the loading of a static pressure of 60% UCS. We selected four typical stress fields in the loading process and analyzed their changes, as shown in Figure 26. From Figure 27a, it can be seen that TSZ is developed at the upper left and lower ends of the bi-flaws, and the upper left corner of the rock. Figure 27b shows that the TSZ at the upper left fissure end expands diagonally to form an SSZ, while the TSZ at the lower end of the bi-flaws continues to develop on both sides. In Figure 27c, the TSZ at the lower part of the specimen is largely formed. In Figure 27d, the TSZ in the upper left corner also extends horizontally into a tensile crack.

## 5. Strain Field Evolution Laws

### 5.1. Effects of Axial Pressure on the Strain Field Evolution Laws of Specimens with 0° Flaw

**Figure 28:**
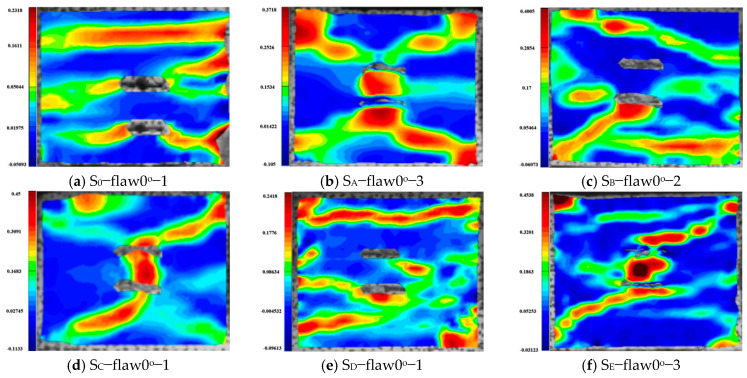
Maximum principal strains during the damage of granite specimens containing 0° parallel double fissures under different combinations of dynamic and static loading.

The fracture of rock contains the process of crack initiation, propagation and coalescence. The damage evolution process is very complex and has very important engineering significance. Through DIC technology, the stress field variation characteristics of fractured rock after failure under dynamic and combined loadings were analyzed [46,47]. The maximum principal strains of 0° specimens under different axial pressures are given in Figure 28, where representative strains from each group were selected for analysis. As can be seen from the figures, the damage patterns of the 0° inclined specimens under different axial pressures are still very different. Under a combined, dynamic and static loading of 0% UCS, the granite with parallel double fissures breaks with three TSZ running through the two flaws of the specimen. The uppermost TSZ shows a significant increase in tensile strain values, eventually presenting significant tensile damage.

Under a combined dynamic and static loading of 10% UCS, the damage pattern of the granite with parallel double fissures changes to two diagonal TSZ through the upper and lower flaws. A main tensile crack through one side is produced between the parallel bi-flaws, and a larger TSZ is produced in the middle of the bi-flaws. Under a combined dynamic and static loading of 20% UCS, a diagonal SSZ through the specimen appears in the upper part of the granite containing parallel double fissures. The lower part of the fissure shows a composite strain zone with shear and tensile strains interspersed.

The granite with parallel bi-flaws shows an obvious “X”-shaped SSZ developing in the center of the fissure under a combined dynamic and static loading of 30% UCS. Under a combined dynamic and static loading of 50% UCS, the upper part of the granite with parallel double fissures has a TSZ through the fissures in the parallel loading direction. In the lower part, there is a composite tensile-shear strain zone through the flaws and a large local TSZ at the lower right end. Under a combined, dynamic and static loading of 60% UCS, the damage to granite with parallel double fissures is caused by a primary SSZ through the lower fissure and a secondary SSZ connecting the upper fissure to the upper right end. A large TSZ is located in the middle of the parallel fissure and at the upper left end of the specimen, with the middle TSZ penetrating the left end and two TSZ at each of the top and bottom of the specimen.

### 5.2. Effects of Axial Pressure on the Strain Field Evolution Laws of Specimens with a 45° Flaw

**Figure 29:**
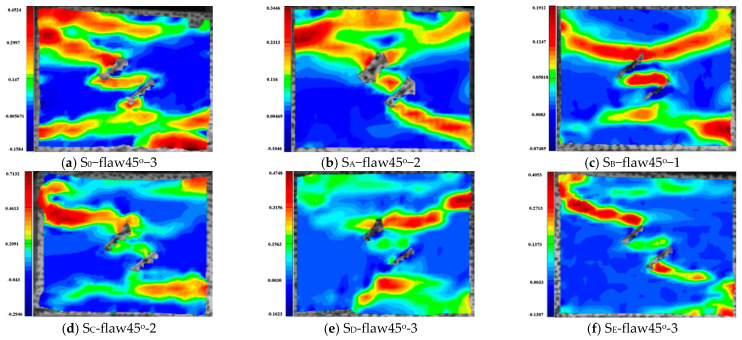
Maximum principal strain clouds during the damage of granite specimens containing 45° parallel double fissures under different combinations of dynamic and static loading.

Figure 29 shows the maximum principal strains of the 45° inclined angle specimens with parallel double fissures damaged. The specimens with a 45° all develop an inverse SSZ during the damage of the dynamic and static loading. At the end of the parallel double fissure, the penetration forms a TSZ parallel to the direction of stress loading. Under the static and dynamic combined load of 0% UCS, the granite containing parallel double fissures produces two parallel TSZs with significant tensile strain values along the upper left and lower right corners. These zones penetrate both ends of the specimen and intersect with the diagonal inverse SSZ, causing damage to the specimen. Under the combined, dynamic and static loading of 10% UCS, the granite containing parallel double fissures produces two parallel TSZ from the upper left to the lower right. These zones penetrate both ends of the specimen and intersect with the diagonal inverse SSZ, causing damage to the specimen.

In addition to the diagonal SSZ, a horizontal TSZ is generated at the upper end of the granite, which passes through the specimen in the parallel loading direction, causing large deformation damage at the intersection. Under a combined, dynamic and static loading of 20% UCS, a horizontal TSZ is generated in the upper and lower parts. The TSZ is formed at the inner end towards the horizontal bi-flaws due to the penetration, in which an inverse SSZ may also exist. In addition to the inverse SSZ through the diagonal of the specimen, a smaller SSZ along the fissure direction intersects the inverse SSZ, which extends to the end of the specimen and causes damage.

In the case of 30% UCS, the SSZ is also smaller in the direction of the fissure and extends to the end of the specimen. In the upper part of the flaw end, a smaller SSZ in the flaw direction is formed, while a TSZ appears in the lower part of the flaw end. At the same time, there is also a TSZ in the upper part of the granite, causing damage. In addition to them, under the combined dynamic-static loading of 50% UCS, one TSZ appeared above and below the crack tip and gradually expanded in the loading direction. Under a combined, dynamic and static loading of 60% UCS, granite damage in parallel double specimens is produced by the typical SSZ in the diagonal direction. At the end of the contact between the specimen and the bar, there is an enhanced strain signal and a horizontal TSZ in the upper part of the granite.

### 5.3. Effects of Axial Pressure on the Strain Field Evolution Laws of Specimens with a 90° Flaw

**Figure 30:**
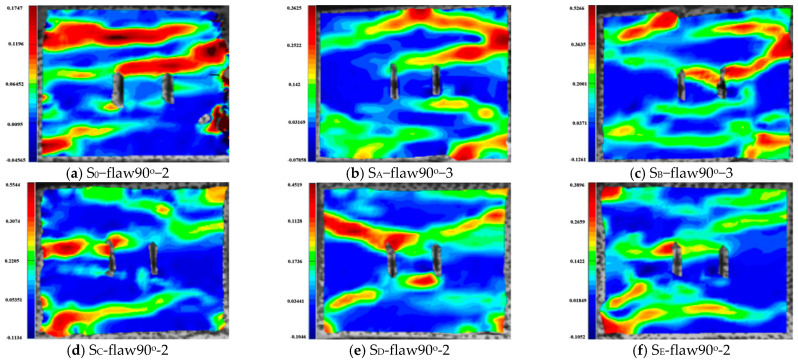
Maximum principal strain clouds during the damage of granite specimens containing 90° parallel double fissures under different combinations of dynamic and static loading.

Figure 30 shows the maximum principal strains at the damage site of the 90° inclined angle specimen with parallel double fissures under different axial pressures. The two primary TSZ and one secondary TSZ were formed in the granite containing parallel bi-flaws under a combined, static and dynamic loading of 0% UCS. One of the primary TSZs appears through the upper end of the parallel bi-flaws, and the extension of the three primary strain zones damages the granite. Under a combined static and dynamic loading of 10% UCS, the upper part generates a tensile crack through the upper end of the flaws, which is related to a tensile crack in the upper part of the granite. The tensile crack at the upper end of the parallel double fissure penetrates the TSZ at the right end, while a penetrating SSZ is generated at the lower end of the parallel double fissure.

Two TSZ were generated at the lower part of the granite under a combined static and dynamic loading of 20% UCS. One is approximately parallel to the loading direction through both ends of the granite, and the other develops through the end of the parallel double fissure. The upper part of the specimen shows a significant diagonal development of SSZ, which also develops through the end of the parallel double fissure. Under the combined loading of 30% UCS, two SSZ were generated in the upper left and lower left parts of the specimen. Both zones develop similarly to the “X”-type SSZ, which finally causes damage to the specimen. Additionally, under the combined static and dynamic loading of 50% UCS, two SSZ are generated through the upper and lower ends of the parallel double fissures. In the lower part of the specimen, a TSZ is generated under the combined static and dynamic loading of 60% UCS. At the same time, an SSZ develops diagonally from the ends of the granite towards the end of the flaw in the upper part.

### 5.4. Fractal Analysis

According to the mass fractal model of rock fragment distribution of rock fragments established by Mandelbrot and other scholars, the fractal dimension D can be obtained according to the mass-frequency relationship of the screening test. The distribution equation of rock fragments under impact load is:

(3) $$M\left(x\right)/M_{T}={\left(x/x_{m}\right)}^{3-D}$$

In the formula, $M\left(x\right)$ and $M_{T}$ represent the total mass of the fragments and the cumulative mass under the sieve, respectively; x and $x_{m}$ represents the particle size and maximum particle size of the fragment; D is the fractal dimension of fragment distribution.

By taking the logarithm of both sides of the above formula at the same time, we can obtain:

(4) $$\mathrm{lg}\left[M\left(x\right)/M_{T}\right]=\left(3-D\right)\mathrm{lg}\left(x/x_{m}\right)$$

**Figure 31:**
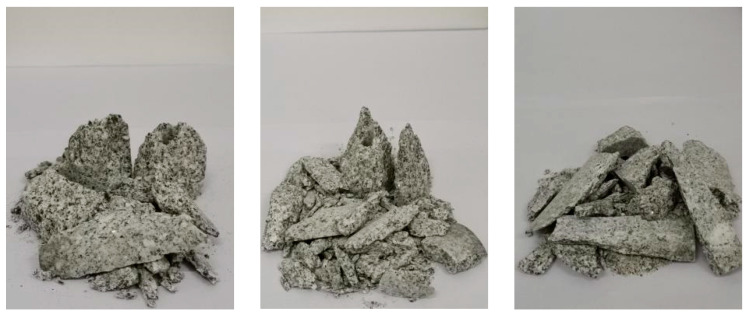
Fracture morphology of disturbed samples after impact failure.

**Figure 32:**
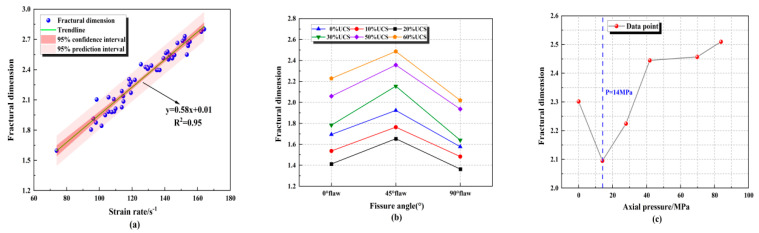
The correlation between fractural dimension and (**a**) strain rate, (**b**) fissure angle and (**c**) axial pressure.

The fractural dimension of rock specimens could directly and quantitatively reflect the degree of rock fracture [48]. The larger the dimension of the specimen, the more fragments; the smaller the volume, and the higher the degree of fragmentation. In order to analyze the fractural characteristics, the pieces were collected for sieving after the impact was completed (Figure 31). In order to quantitatively analyze the failure state under different conditions, the fractural dimensions were calculated, and the relationship between the fractural dimension and the strain rate, the axial pressure and the fissure angle were discussed. It can be seen from Figure 32a that the fractural dimension increased with the increase in the strain rate, which showed a good linear correlation. In the test, the granite strain rate increased from 73.93 s^−1^ to 164.08 s^−1^, and the fractural dimension increased from 1.5963 to 2.8014. As shown in Figure 32b, the fractural dimension is closely related to the fissure angle. The fractural dimension showed the change law of decreasing and then increasing. When the angle is 90°, the dimension is the smallest, and at 45°, it reaches the maximum. Figure 32c showed that the fractural dimension first decreased and then increased with the increase in axial pressure, but it is slightly higher when the axial pressure is 0% UCS, which is opposite to the dynamic strength, which illustrates that in engineering practices, bridge angle and fractural dimension affect the rock fragmentation degree. Therefore, appropriately increasing the angle of the excavation fissure and reasonably utilizing the natural in situ stress will help to improve the efficiency of rock excavation and rock breakage to some extent.

## 6. Discussion

The final damage pattern of granite specimens at the same fracture inclination is influenced by the magnitude of the applied static load: (1) zero degree parallel bi-flaws. The granite with parallel double fissures at the combined dynamic and static loading of 0° produces three tensile strain cracks parallel to the loading direction, which eventually leads to tensile damage of specimens. In contrast, a distinct SSZ appears with combined dynamic and static loading, with typical tensile-shear damage at the combined dynamic and static loadings of 20% and 50% under uniaxial compression, and shear damage at the combined dynamic and static loadings of 10%, 30% and 60%. Significantly, the shear damage pattern is dominant at the combined dynamic and static loadings of 10%, 30% and 60% under uniaxial compression. (2) forty-five degree parallel bi-flaws. At a loading of 0–60%, the change in axial pressure has little effect on the damage mode of the specimens with 45° cracks, which basically form an inverse SSZ along the end of the LAW. With the increase in axial pressure, this shear mode becomes increasingly significant, and there is a tendency to transition from shear-tension damage mode to shear damage mode. (3) ninety-degree parallel bi-flaws. The shear strain zone and the length and number of shear cracks increase with increasing axial pressure, and there is a tendency to transition from tensile to shear-tensile damage modes.

## 7. Conclusions

Analysis of the damage process of fractured granite under combined dynamic and static loading by the DIC technique has obtained many details and processes of damage that cannot be observed by the naked eye, providing powerful approaches to understanding the damage of rock under dynamic and static loading. This is of great significance for further study of the mechanism of rockburst and the prevention of rockburst. However, although disturbance excavation is the most common way of deep rock mass engineering, it provides a new idea for the efficient crushing of rock mass if the high storage energy in rock mass can be effectively utilized to induce rock breaking. This study can be summarized as follows:

1. As the angle of inclination increases, the peak stress increases, and it can be concluded that the external impact resistance of the rock is minimized when the inclination angle of the fissure within the rock is approximately 45°. Changes in the angle produce a certain degree of weakening of the dynamic and combined strengths. Changes in the inclination angle affect the distribution of the internal stress field of the rock during the impact of the specimen, which is macroscopically manifested by the weakening of the dynamic and combined strengths.
2. The peak strain and dynamic modulus of elasticity, affected by the weakening of the inclination angle, are similar to the strength. The peak strain shows an overall trend that increases and then decreases with the increase in axial pressure. The inflection point of the trend occurs at an axial pressure of 27.9 MPa (20% UCS). The overall axial pressure at 0~50% UCS increases step by step.
3. The analysis of the maximum principal strains of the specimens during the damage shows that the specimens all eventually evolved from tensile strain to shear damage. Significantly, the parallel fracture specimens with an inclination angle of 45° show an inverse shear strain zone at different axial pressure conditions, which does not appear in the 0° and 90° specimens. The damage patterns of the 0° and 90° inclined specimens at different axial pressures differed significantly. The 45° inclined specimens show an inverse flank shear strain zone at combined dynamic and static loading damage, while the 0° and 90° inclined specimens show an “X”-shaped shear strain zone at different axial pressures.
4. Both the axial pressure and flaw inclination angle affect the fractal dimension of double-flawed rocks. With the increase in fracture angle, the fractal dimension first increases and then decreases, reaching its maximum at 45°. The fractural dimension increases with the increase in the strain rate, which showed a good linear correlation.

## Figures and Tables

**Table 1:** Physical parameters and experimental results of granite specimens under combined static and dynamic loads.

Specimen Number	Length /mm	Height /mm	Thickness /mm	Fissure Angle/°	Axial Pressure/MPa	Dynamic Strength/MPa	Combined Strength/MPa	Peak Strain/10^−3^	Strain Rate/s^−1^
S_0_-flaw0°-1	45.37	45.1	20.2	0	0% UCS	155.60	155.60	8.83	109.16
S_A_-flaw0°-2	45.24	45.14	20.53	0	10% UCS	196.50	210.50	6.62	98.37
S_B_-flaw0°-3	45.08	45.4	19.88	0	20% UCS	181.63	209.53	5.17	73.93
S_C_-flaw0°-1	45.04	45.37	19.86	0	30% UCS	189.45	231.35	5.53	114.61
S_D_-flaw0°-1	45.26	45.29	20.13	0	50% UCS	166.90	236.70	4.28	140.65
S_E_-flaw0°-1	45.09	45.33	20.20	0	60% UCS	149.32	233.12	4.04	131.82
S_0_-flaw45°-1	45.27	45.22	20.28	45	0% UCS	134.79	134.79	5.30	153.50
S_A_-flaw45°-2	45.30	45.15	20.27	45	10% UCS	161.25	175.25	6.54	139.22
S_B_-flaw45°-2	45.16	45.26	20.24	45	20% UCS	132.27	160.17	5.46	144.60
S_C_-flaw45°-2	45.04	45.43	20.31	45	30% UCS	132.68	174.58	5.37	154.26
S_D_-flaw45°-1	45.16	45.25	20.18	45	50% UCS	102.26	172.06	3.37	164.08
S_E_-flaw45°-1	45.37	45.34	20.29	45	60% UCS	106.25	190.05	3.57	162.34
S_0_-flaw90°-1	45.21	45.29	20.31	90	0% UCS	141.32	141.32	6.07	136.89
S_A_-flaw90°-2	45.06	45.37	20.36	90	10% UCS	179.34	193.34	7.13	105.62
S_B_-flaw90°-2	45.05	45.3	20.26	90	20% UCS	159.76	187.66	5.11	119.33
S_C_-flaw90°-3	45.22	45.13	20.30	90	30% UCS	170.19	212.09	5.73	142.65
S_D_-flaw90°-1	45.25	45.14	20.21	90	50% UCS	145.30	215.10	4.38	121.70
S_E_-flaw90°-2	45.15	45.27	20.42	90	60% UCS	111.07	194.87	3.54	147.74

## Data Availability

Data sharing not applicable.
